# miR186 suppresses prostate cancer progression by targeting Twist1

**DOI:** 10.18632/oncotarget.8887

**Published:** 2016-04-21

**Authors:** Xian Zhao, Yanli Wang, Rong Deng, Hailong Zhang, Jinzhuo Dou, Haihua Yuan, Guofang Hou, Yuzhang Du, Qin Chen, Jianxiu Yu

**Affiliations:** ^1^ Department of Biochemistry and Molecular Cell Biology, Shanghai Key Laboratory of Tumor Microenvironment and Inflammation, Shanghai Jiao Tong University School of Medicine, Shanghai 200025, China; ^2^ State Key Laboratory of Oncogenes and Related Genes, Shanghai Jiao Tong University School of Medicine, Shanghai 200025, China; ^3^ Department of Pathophysiology, Key Laboratory of Cell Differentiation and Apoptosis of Chinese Ministry of Education, Shanghai JiaoTong University School of Medicine, Shanghai 200025, China; ^4^ Institute of Oncology & Department of Oncology, Shanghai 9th People's Hospital, Shanghai Jiao Tong University School of Medicine, Shanghai 200025, China

**Keywords:** prostate cancer (PCa), microRNA (miRNA), metastasis, miR186, Twist1

## Abstract

Prostate cancer (PCa) is the second leading cause of cancer-related deaths in north American men, and most its related deaths are due to advanced and metastatic PCa. However, the molecular mechanisms underlying PCa progression are still unclear. Here we use a pair of prostate cell lines P69/M12, which have the same genetic background and the highly metastatic cell line M12 is a subline derived from P69, to identify the pathogenesis of PCa. We find that a key miRNA--miR186 is significantly reduced in M12 compared to that in P69. Further, we validate that miR186 is also downregulated in human PCa specimens, most significantly in the metastatic patient specimens. The low miR186 expression is correlated with poor patient survival. Through knockdown or overexpression of miR186 in PCa cell lines, we discover that miR186 strongly inhibits cell motility, invasive, soft-agar colony formation, 3D culture growth and vasculogenic mimicry (VM) formation capacity, as well as the epithelial-to-mesenchymal transition (EMT) process by downregulation of its target Twist1. Moreover, the inverse relationship between the expression levels of miR186 and Twist1 is confirmed *in vivo* tumor metastasis experiment and clinical specimens. Taken together, our findings demonstrate an important role of miR186/Twist1 axis in the regulation of PCa progression, suggesting a potential application of miR186/Twist1 in PCa treatment.

## INTRODUCTION

Prostate cancer (PCa) has become the most frequently diagnosed cancer and the second leading cause of cancer-related deaths in North American men [1, 2]. Over 30,000 men die each year from prostate cancer-related illness in the United States [3]. The number of afflicted men is increasing rapidly as the population of males over the age of 50 grows worldwide. Nearly one third of PCa patients have micrometastatic disease at the time of presentation, which is largely undetected due to a lack of sensitive biomarkers. Advanced PCa can spreads mainly to bone, also lung or other parts of the body, and metastatic PCa remains the main cause of PCa-related death in men. Finding strategies for the prevention of prostate cancer is a crucial medical challenge.

Metastasis is a multistep process by which primary tumor cells undergo Epithelial–mesenchymal transition (EMT), invade adjacent tissue, enter the blood stream, survive in the circulation, extravasate into the surrounding tissue and finally form clinically detectable metastases [4]. EMT is a process in which adherent epithelial cells shed their epithelial traits and acquire mesenchymal properties, such as increased potential for motility invasion/metastasis and resistance to chemotherapy [5, 6]. A group of transcription factors have been demonstrated to be capable of orchestrating EMT program in cancer progression, such as Snail (SNAI1), Slug (SNAI2), ZEB2 (SIP1), ZEB1 and Twist1 [7].

Twist1, a highly conserved basic helix–loop–helix transcription factor, is a key factor in promoting metastasis of cancer cells [7]. Twist1, acting as a repressor of E-cadherin, induces EMT and endows cells with the enhanced migratory and invasive capabilities necessary for metastasis, which is one of the most important processes in cancer progression [8]. In PCa, Twist1 is upregulated and its expression level is positively associated with Gleason grading [9]. Twist1 confers taxol resistance to PCa cells [10] and promotes PCa progression through EMT. The increased expression level of Twist1 in PCa patient is correlated with poor outcome and shorter patient survival, indicating it plays important role in PCa progression and may be a potential therapeutic target.

MicroRNAs (miRNAs) play critical roles in a variety of biological processes, including cell proliferation, development, differentiation, apoptosis, metabolism and immunity [11–13]. Accumulating evidence has demonstrated that miRNAs have also roles in EMT and metastasis. For example, miR-9 may initiate EMT and promote breast cancer cell metastasis by directly targeting E-cadherin [14]; the miR-200 family members and miR-205 inhibit EMT and cell invasiveness by targeting EMT transcription factors ZEB1 and ZEB2 [15–17].

In this study we found that miR186 was downregulated inmalignant PCa cell lines and clinical PCa specimens, and we experimentally demonstrated that miR186 maintained the epithelial phenotype and reduced migration and invasion, soft-agar colony formation, vasculogenic mimicry (VM) formation capability of human PCa cells through targeting Twist1, suggesting that miR186 might serve as a tumor-suppressive miRNA in the development and progression of PCa. Most importantly, one-way ANOVA analysis showed that the miR186 expression levels were statistically significant differences in subgroups clinical grade (P < 0.001), Cleason (P < 0.001), TNM stage (P < 0.01), indicating that the decreased miR186 expression contributed to PCa progression and might represent a prognostic biomarker for PCa.

## RESULTS

### miR186 is downregulated in a malignant PCa cell line M12

**Figure 1:**
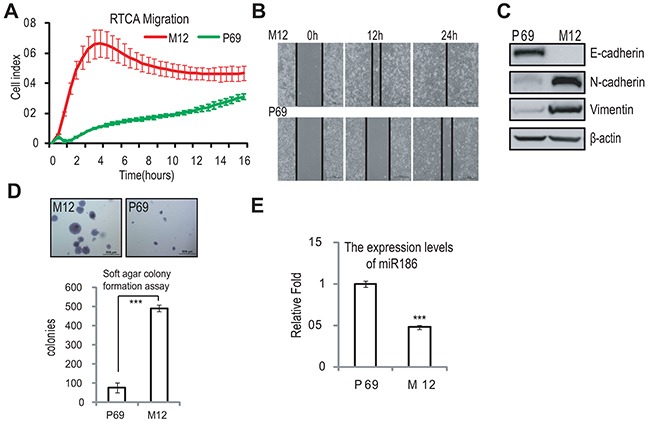
miR186 is downregulated in malignant prostate cancer cell line M12 **A.** RTCA (real time cell analysis) monitoring of cell migration by using the xCELLigence system RTCA-DP. P69 and M12 cells were seeded to a CIM-Plate and subjected to a dynamic migration assay lasting for 16 h. The migration curve slope was shown as histogram. Error bars indicate ±SD. **B.** Wound-healing assays for M12 and P69 cells. Serum was withdrawn before analysis to avoid effect of cell proliferation. Representative pictures were taken at indicated times. Experiments were performed three independent times. **C.** Immunoblotting of epithelial and mesenchymal markers in P69 and M12 cells. **D.** Soft agar colony formation assays for M12 and P69 cells. The culture medium containing 5% FBS with 0.35% agar was layered onto the base. The photographs were taken (upper panel) and the number of colonies was scored (low panel). The same scale bar (500 μ m) was used in all images. Each value represents the mean ±SEM of three independent experiments with triplicates each. An unpaired (equal variance) t-test was performed, *P*-values of < 0.001 (***). **E.** Real-time PCR analysis for the expression levels of miR186 in P69 and M12 cells. U6 was used as internal control. The experiments were performed at least three independent times, and error bars indicate ± SEM, *P*-values of < 0.001 (***).

To investigate the potential mechanism of PCa progression, we used a pair of PCa cell line P69/M12, which have the same genetic background and M12 is a subline derived from P69 *via* selection in nude mice, undergoing DNA damage, transformation, immune escape *in vivo* [18–20]. In order to make sure M12 having occurred transformation, we firstly used the xCELLigence RTCA-DP System [20, 21] to real-time monitor the migration capacity of P69 and M12 (Figure 1A). P69 displayed a relative flat curve in cell index of migration, whereas M12 exhibited a more sharp one, and the wound healing assays also indicated that M12 had high motility capacity compared to that P69 (Figure 1B). In view of cell motility is always associated with EMT, so we examined the EMT marker levels in these two cell lines. M12 exhibited the high levels of mesenchymal markers including N-cadherin and Vimentin; in contrast, P69 showed the high levels of epithelial marker E-cadherin (Figure 1C). To further assess the ability for transformation, the soft agar colony formation assays were performed. The result revealed that M12 endowed with high anchorage-independent growth abilities compared to that of P69, as evidenced by the numbers as well as the size of formed colonies measured (Figure 1D). Therefore, this model of M12/P69 cell lines can be used in the study of PCa progression. To investigate which miRNA is involved in the progression from P69 to M12, we performed the real-time PCR and found that miR186 was significantly downregulated in M12 compared to that in P69 (Figure 1E).

### MiR186 suppresses PCa progression

**Figure 2:**
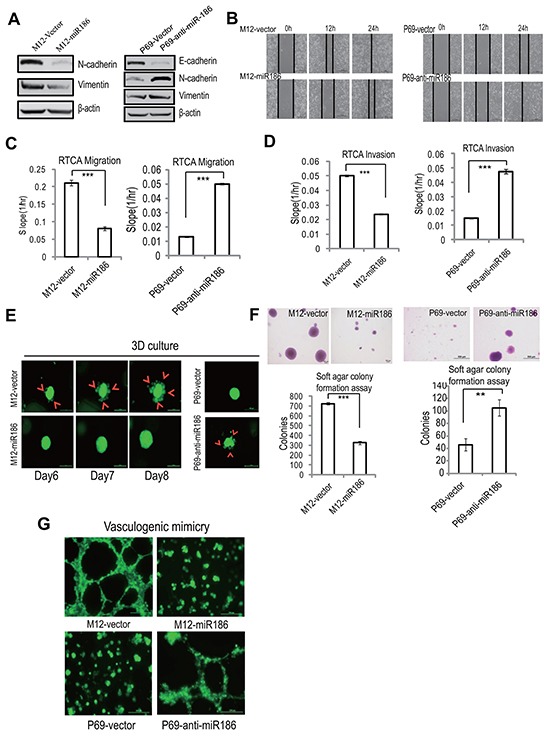
miR186 suppresses PCa cell progression **A.** Immunoblotting of epithelial and mesenchymal markers in M12-vector and M12-miR186 or P69-vector and P69-anti-miR186 cells. **B.** Wound-healing assays for M12-vector and M12-miR186 or P69-vector and P69-anti-miR186 cells. Serum was withdrawn before analysis to avoid effect of cell proliferation. Representative pictures were taken at indicated times. Experiments were performed three independent times. **C–D.** RTCA monitoring of cell migration (C) or invasion (D). M12-vector and M12-miR186 or P69-vector and P69-anti-miR186 cells were seeded into a CIM-Plate without or with pre-coated matrigel (1:40) and subjected to a dynamic analysis lasting for 48 h, respectively. The migration or invasion slope was shown as histogram. Error bars indicate ±SD, *P*-values of < 0.001 (***) (also See Supplementary Figure S1B–S1C). **E.** 3D culture growth assay for M12-vector and M12-miR186 or P69-vector and P69-anti-miR186 cells. Representative images of cell morphology in extracellular matrix were taken. **F.** Soft agar colony formation assays for M12-vector and M12-miR186 or P69-vector and P69-anti-miR186 cells. The culture medium containing 5% FBS with 0.35% agar was layered onto the base. The number of colonies was counted for each well of six-well plates (low panels), the sizes of colonies was shown (upper panels). The experiments were repeated by three independent times with triplicates each. Error bars indicate ±SEM, *P*-values of < 0.001 (***). **G.** VM assay for M12-vector and M12-miR186 or P69-vector and P69-anti-miR186 cells. Representative images of vasculogenic networks in extracellular matrix were taken.

To assess the role of the dyregulated miR186 in PCa cells, we tested whether the ectopic expression or the inhibition of miR186 expression can block or accelerate the PCa progrssion, respectively. MiR186 was introduced to M12, or silenced with antisense inhibitor (anti-miR186) in P69. The efficiency of ectopic expression in M12 was determined by real-time PCR, the miR186 level was increased up to 2 fold similar to that in P69 (Supplementary Figure S1A). The ectopic expression of miR186 in M12 markedly downregulated N-cadherin and Vimentin (mesenchymal markers) (Figure 2A, left panel); on the contrary, the silencing of endogenous miR186 in P69 upregulated N-cadherin, Vimentin and decreased E-cadherin (Figure 2A, right panel). These results suggest that miR186 can inhibit the EMT progression. Next, to determine the effects of miR186 on cell migratorycapacity, we employed the wound healing assays (Figure 2B) and RTCA migration assays [21] (Figure 2C and Supplementary Figure S1B). The results showed that the cell migratory capacity was greatly inhibited by overexpression of miR186 in M12, on the contrary, enhanced by silencing of miR186 in P69, respectively. The effect of miR186 on the invasive capacity was evaluated by xCELLigence System using CIM-Plate pre-coated with matrigel [21]. As expected, ectopic expression of miR186 in M12 strikingly decreased the number of cells penetrated into matrigel, while silencing of miR186 in P69 promoted cell aggressiveness (Figure 2D and Supplementary Figure S1C). Furthermore, by using the method of 3D cultures on extracellular matrix to mimic *in vivo* conditions, we found that M12 grew extensively inside the semisolid collagen gel and displayed an elongated or scattered morphology, showing their ability to invade into extracellular matrix. However, ectopic expression of miR186 abolished cell penetrating into the matrigel, instead, cells grew into tight colonies and only a small percentage of cells showed invasive ability in the matrigel (Figure 2E, left panels). In contrast, knockdown of miR186 in P69 triggered cells invading into the matrigel (Figure 2E, right panels). In attempt to examine the effect of miR186 on tumorigenesis, the soft-agar colony formation assays were preformed. Ectopic expression of miR186 in M12 dramatically decreased while silencing of miR186 in P69 increased the soft-agar colony formation in numbers and sizes (Figure 2F). We also assessed whether miR186 influences vasculogenic mimicry (VM) formation of PCa cells. M12 was able to rapidly form vasculogenic networks on the matrigel, which was destroyed by ectopic expression of miR186 (Figure 2G). P69 could only accumulate in clumps on the matrigel, whereas the pipe-like structures was observed when miR186 was knocked down (Figure 2G). Collectively, above data demonstrate that miR186 playstumor suppressive roles in PCa progression by inhibiting EMT, migration, invasion, anchorage-independent growth, 3D culture growth and VM formation.

### Twist1 is a direct target of miR186 in PCa

**Figure 3:**
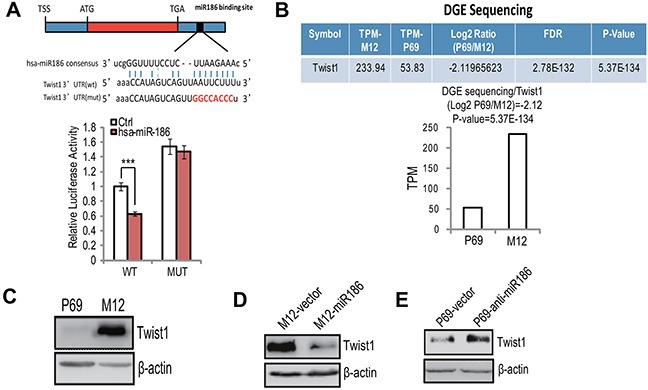
Twist1 is a direct target of miR186 in PCa **A.** The putative miR186 binding site in Twist1 3′-UTR, as predicted by miRanda (upper panel); luciferase activity assays with the wild-type 3′UTR or mutated 3′UTR of Twist1 cotransfeced with Control or miR186 into 293T. The renilla luciferase values were normalized to the firefly luciferase activity and plotted as relative luciferase activity. ***, P<0.001 (low panel). **B.** The mRNA levels of Twist1 in P69 and M12 cells were analyzed by DGE sequencing. *P*-values of < 0.001 (***). **C–E.** Western blotting analysis for Twist1 in P69 and M12 (C), M12-vector and M12-miR186 (D), or P69-vector and P69-anti-miR186 cells (E).

To investigate the key target(s) involved in PCa progression by miR186, we searched putative target genes using bioinformatics prediction program miRanda-mirSVR. Among many candidates, we focused on the transcription factor Twist1 because of its high score (mirSVR score: -1.1393) and its function related with EMT and metastasis. In order to determine that miR186 directly binds the Twist1 3′-UTR, we performed the luciferase reporter assay. The miR186 significantly repressed the activity of the Twist1-3′-UTR luciferase reporter construct containing a wild-type but not a mutated miR186 binding site (Figure 3A). Through high-throughput sequencing of digital gene expression (DGE) and Western blotting analysis, we found that the expression level of Twist1, which was higher in M12 than those in P69 (Figure 3B–3C), was inversely correlated with miR186 (Figure 1E). Most importantly, the protein level of Twist1 was indeed reduced drastically in M12-miR186 cells (Figure 3D) but elevated in P69-anti-miR186 cells (Figure 3E). These results indicate that miR186 directly suppresses Twist1 by targeting Twist1 3′-UTR.

### Twist1 mimics miR186-mediated phenotypes in PCa cells

**Figure 4:**
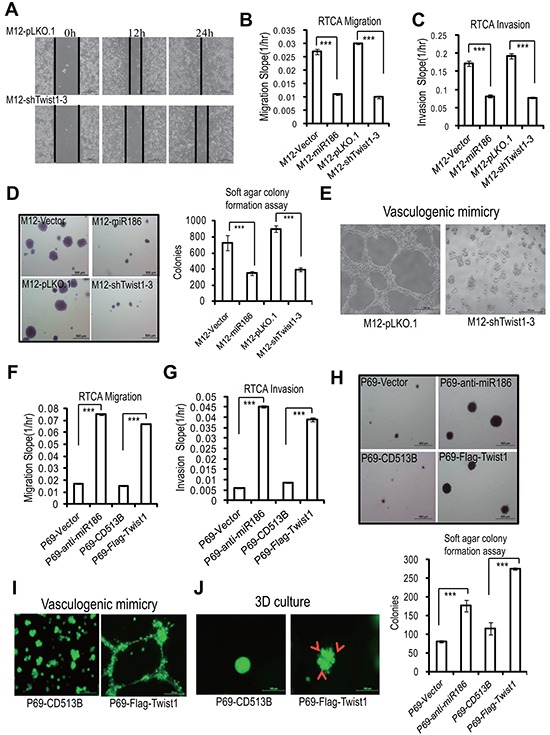
Twist1 mimics miR186-mediated phenotypes in PCa cells **A.** Wound-healing assays for M12 cells stably transfected with pLKO.1 vector or shTwist1-3. Serum was withdrawn before analysis to avoid effect of cell proliferation. Representative pictures were taken at indicated times. Experiments were performed three independent times. **B–C, F–G.** RTCA monitoring of cell migration (B, F) or invasion (C, G) using the xCELLigence system. Stable M12 group (M12-vector, M12-miR186, M12-pLKO.1 and M12-shTwist1-3 in B–C) or P69 group (P69-vector, P69-anti-miR186, P69-CD513B, and P69-Twist1 in F–G) cells were seeded to a CIM-Plate without or with pre-coated matrigel (1:40) and subjected to a dynamic analysis lasting for 48 or 72 h, respectively. The migration or invasion slope was shown as histogram. Error bars indicate ±SD, P-values of < 0.001 (***). (also see Supplementary Figure S2B–S2C, S2D–S2E). **D, H.** Soft agar colony formation assays for above stable M12 group (D) and P69 group (H) cells. The culture medium contains 5% FBSwith 0.35% agar and layered onto the base. Representative images of colonies were taken. The same scale bar (500 μm) was used in all images. The number of colonies was counted for each well of six-well plates. The experiments were repeated by three independent times with triplicates each. Error bars indicate ±SEM, P-values of < 0.001 (***). **E.** VM assays for M12-pLKO.1 and M12-shTwist1-3 cells. Representative images of vasculogenic networks in extracellular matrix were taken. **I–J.** VM assays (I) and 3D culture growth assays (J) for P69-CD513B, and P69-Twist1 cells. Representative images of vasculogenic networks or cell morphology in extracellular matrix were taken and shown.

To clarify the roles of Twist1 in PCa, we performed the reverse function gain/loss experiments. M12 were transfected with a lentiviral-shRNA specific for Twist1 (pLKO-shTwist1-3), leading to almost complete abolishment of Twist1 expression (Supplementary Figure S2A, lane 4). Knockdown of Twist1 significantly reduced cell migration (Figure 4A–4B & Supplementary Figure S2B), invasion (Figure 4C & Supplementary Figure S2C), anchorage-independent growth (Figure 4D) and VM formation (Figure 4E), which all were similar to the phenotypies mediateded by overexpression of miR186. On the contrary, ectopic expression of Twist1 in P69 cells resulted in enhancement of migration (Figure 4F & Supplementary Figure S2D), invasion ability (Figure 4G & Supplementary Figure S2E), anchorage-independent growth (Figure 4H), VM formation (Figure 4I) and 3D culture growth (Figure 4J), as like phenocopying those in knockdown of miR186 in P69 (P69-anti-miR186 cells). Above results demonstrated that miR186 and Twist1 execute opposite effects during PCa progression.

### miR186 functions by downregulation of Twist1 in PCa cells

**Figure 5:**
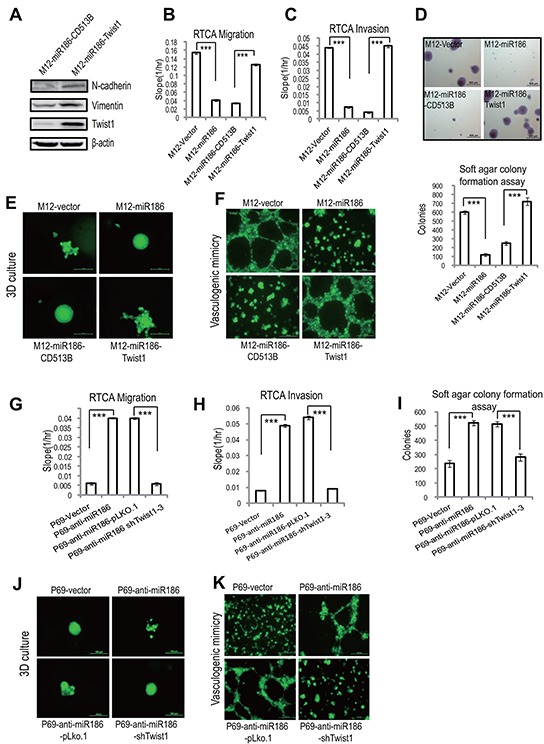
miR186 functions by downregulation of Twist1 in PCa cells **A.** Immunoblotting of epithelial and mesenchymal markers in M12-miR186-Vector and M12-miR186-Twist1 cells. **B–C, G–H.** RTCA monitoring of cell migration (B, G) or invasion (C, H). M12 group (M12-Vector, M12-miR186, M12-miR186-CD513B and M12-miR186-Twist1 in B–C) or P69 group (P69-Vector, P69-anti-miR186, P69-miR186-pLKO.1 and P69-anti-miR186-shTwist1-3 in G–H) cells were seeded into a CIM-Plate without or with pre-coated matrigel (1:40) and subjected to a dynamic analysis lasting for 48 or 72 h, respectively. The migration or invasion slope was shown as histogram. Error bars indicate ±SD, *P*-values of < 0.001 (***). (also see Supplementary Figure S3B–3C, 3F–3G). **D, I.** Soft agar colony formation assays for above stable M12 group (D) and P69 group cells (I). The culture medium containing 5% FBS with 0.35% agar and layered onto the base. Representative images of colonies were taken (top panel) and the number of colonies was counted for each well of six-well plates and shown (bottom panel). The experiments were repeated by three independent times with triplicates each. Error bars indicate ±SEM, *P*-values of < 0.001 (***). **E–F, J–K.** 3D culture growth assays (E, J) and VM assays (F, K) for above stable M12 group (E–F) and P69 group cells (J–K). Representative images of cell morphology and vasculogenic networks in extracellular matrix were taken.

To further investigate whether Twist1 is a direct functional mediator of miR186 and the miR186-associated phenotype can be rescued by Twist1, a series of rescue experiments were carried out. Overexpression of Twist1 in M12-miR186 cells (as shown in Supplementary Figure S3A) abrogated miR186 mediated inhibition of N-cadherin/Vimentin expression (Figure 5A), cell migration (Figure 5B & Supplementary Figure S3B), cell invasion (Figure 5B & Supplementary Figure S3C), cell anchorage-independent growth (Figure 5D), 3D culture growth (Figure 5E) and VM formation (Figure 5F). In parallel, we established stable knockdown of Twist1 by using the lentiviral-shRNA interference in P69-anti-miR186 cells (Supplementary Figure S3D) to evaluate whether it can overcome the effect of miR186 silencing. Indeed, knockdown of Twist1 reversed various phenotypes of P69-anti-miR186 cells, including expressions of EMT markers (Supplementary Figure S3E), cell migration (Figure 5G & Supplementary Figure S3F), cell invasion (Figure 5H & Supplementary Figure S3G), anchorage-independent growth (Figure 5I), 3D culture growth (Figure 5J) and VM formation (Figure 5K). Taken together, the above results suggest that Twist1 is a functional target of miR186 in PCa cells.

### The miR186-Twist1 axis suppresses PCa progression

**Figure 6:**
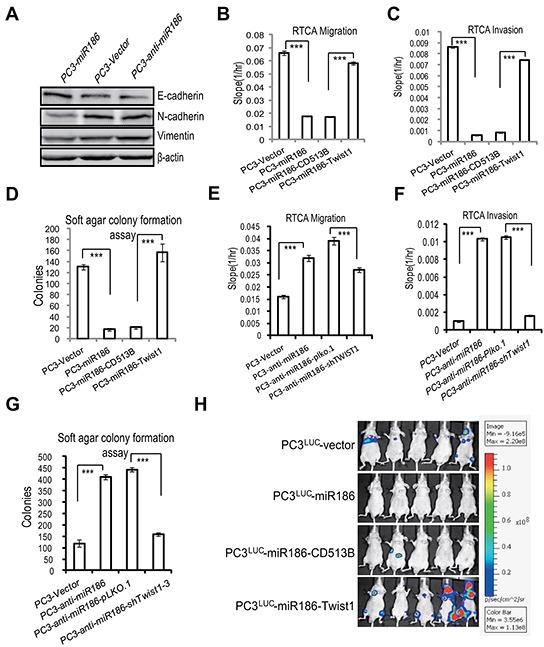
The miR186-Twist1 axis suppresses PCa progression **A.** Immunoblotting of epithelial and mesenchymal markers in PC3-Vector, PC3-miR186 and PC3-anti-miR186 cells. **B–C, E–F.** RTCA monitoring of cell migration (B, E) or invasion (C, F). One group (PC3-Vector, PC3-miR186, PC3-miR186-CD513B and PC3-miR186-Twist1) and another group (PC3-Vector, PC3-anti-miR186, PC3-anti-miR186-pLko.1 and PC3-anti-miR186-shTwist1) cells were seeded into a CIM-Plate without or with pre-coated matrigel (1:40) and subjected to a dynamic analysis lasting for 48 or 96 h, respectively. The migration or invasion slope was shown as histogram. Error bars indicate ±SD, *P*-values of < 0.001 (***). (also see Supplementary Figure S4). **D, G.** Soft agar colony formation assays for above two group cells. The culture medium containing 5% FBS with 0.35% agar was layered onto the base. The number of colonies was counted for each well of six-well plates and shown. The experiments were repeated by three independent times with triplicates each. Error bars indicate ±SEM, *P*-values of < 0.001 (***). **H.** 1.0×10^6^ of stable cell lines PC3^Luc^-Vector, PC3^Luc^-miR186, PC3^Luc^-miR186-Vector and PC3^Luc^-miR186-Twist1 were respectively injected into the left cardiac ventricle of BALB/c nude mice at 7 weeks old by intracardiac injection. Representative bioluminescence images of the micrometastasis was detected by IVIS spectrum image system at 4 weeks after injection and presented visually with various color spots based on different intensity of luminescence.

To further validate that the biological relevance of the miR186-Twist1 axis is a general phenomenon in PCa, similar experiments were performed with a highly malignant and metastatic PCa cell line, PC3. Firstly, overexpression of miR186 in PC3 suppressed whereas knockdown of miR186 promoted the EMT progression (Figure 6A). Secondly, overexpression of Twist1 in PC3-miR186 cells abolished miR186-mediated suppression of cell migration (Figure 6B & Supplementary Figure S4A), cell invasion (Figure 6C & Supplementary Figure S4B), cell anchorage-independent growth (Figure 6D). Thirdly, knockdown of Twist1 in PC3-anti-miR186 cells reversed the enhancement of cell migration (Figure 6E & Supplementary Figure S4C), cell invasion (Figure 6F & Supplementary Figure S4D), cell anchorage-independent growth (Figure 6G) caused by miR186 silencing. All these data from PC3 strengthened our conclusion the miR186-Twist1 axis suppresses PCa cell progression.

Since the androgen receptor (AR) signaling is a key pathway in PCa progression and therapeutic resistance, and PC3 is an AR-negative cell line, we also performed the similar experiments in the AR-positive cell line LNCaP. Through overexpression or knockdown of miR186 in LNCaP cells with the lentiviral vector system, we found that miR186 suppressed EMT (Supplementary Figure S5A) and migration (Supplementary Figure S5B), which are similar to as those in PC3. When knocked down AR in LNCaP cells by siRNA (Supplementary Figure S5C), we found that the levels of miR186 were not affected (Supplementary Figure S5D). Moreover, we knocked down AR in stable cell line LNCaP-Ctrl or LNCaP-anti-miR186 and showed that the miR186-mediated Twist1 inhibition was not influenced by AR (Supplementary Figure S5E). Lastly, we also measured the mRNA levels of AR and PSA in LNCaP-miR186 or LNCaP-Ctrl cells, and found that the mRNA levels of both AR and PSA (AR downstream) were moderately downregulated (Supplementary Figure S5F–S5G). These were probably due to the effect of Twist1 on the AR signaling pathway, as reported that silencing of Twist1 suppresses the AR expression in LNCaP cells through binding to E-boxes in the AR promoter region [22]. Overall, the miR186/Twist1 pathway may also affect the AR signaling pathway, but is independent on the AR pathway. Thus, the miR186-Twist1 pathway seems to work in either negative or positive AR PCa cell line.

All these *in vitro* results prompted us to explore the effects of the miR186-Twist1 axis on tumor metastasis of xenografted mouse model *in vivo*. The malignant PC3*^luc^* cells which were engineered to stably express a firely luciferase were infected with the lentiviral empty vector or carrying miR186 construct. These cells were injected into the left cardiac ventricle of BALB/c nude mice at 7 weeks old by intracardiac injection. The micrometastasis in the whole organs was assessed by IVIS spectrum image system as shown in bioluminescence images and H&E staining. Overexpression of miR186 in PC3^luc^ cells strikingly suppressed tumor metastasis *in vivo*, while re-expression Twist1 in the PC3^luc^-miR186 cells significantly rescued metastasis formation which was inhibited by overexpression of miR186 (Figure 6H & Supplementary Figure S6). Collectively, above results proved that the miR186-Twist1 axis suppresses PCa progression *in vitro* and metastasis *in vivo.*

### Clinical significance of the miR186-Twist1 axis in PCa progression

**Figure 7:**
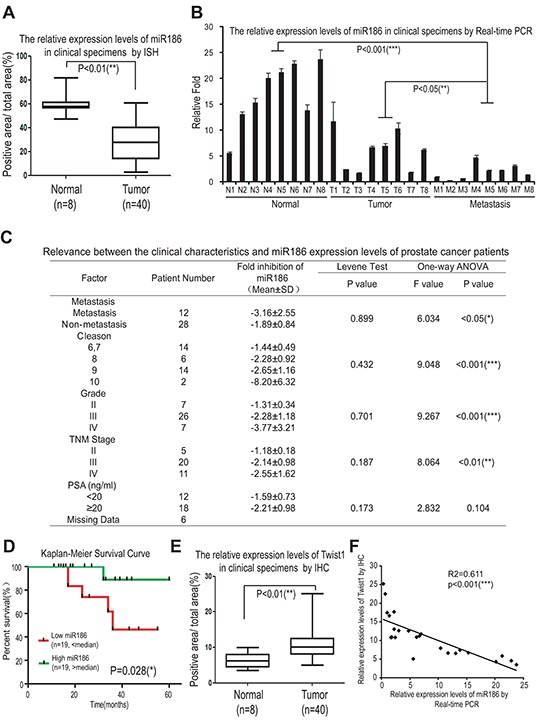
Clinical significance of the miR186-Twist1 axis in PCa progression **A.** Assessment of miR186 levels by *in situ* hybridization in normal prostate tissues (n=8) and prostate carcinomas (n=40). **B.** The miR186 expression levels in clinical specimens were measured by real-time PCR. (‘N’ indicates ‘Nomal’, n=8; ‘T’ indicates ‘Tumor’, n=8; ‘M’ indicates ‘Metastasis’, n=8), U6 was used as a loading control. **C.** The relevance between the clinical characteristics and the miR186 expression levels of prostate cancer patients was analyzed by one-way ANOVA. **D.** The correlation between the miR186 level and survival of patients with PCa was analyzed by Kaplan–Meier analysis. **E.** Assessment of miR186 levels by immunohistochemistry in normal prostate tissues (n=8) and prostate carcinomas (n=40). **F.** The correlation between the levels of miR186 (measured by real-time PCR) and Twist1 (detected by IHC) in clinical PCa specimens was analyzed by Pearson Correlation test and linear correlation and regression.

To investigate the role of miR186 in initiation and progression of human PCa, we first compared the expression levels of miR186 between clinical prostate carcinoma and normal prostate tissues. By *in situ* hybridization (ISH), we showed that the expression levels of miR186 were reduced in prostate tumor specimens (n=40) compared to those of normal prostate tissues (n=8) (Figure 7A and & Supplementary Table S1). The real-time PCR for miR186 was performed to confirm the results of ISH, showing that the expression levels of miR186 were reduced in prostate cancer specimens, especially most significantly in the metastatic patient specimens, compared with paired normal tissues (Figure 7B). Further one-way ANOVA analysis showed that the miR186 expression levels were statistically significant differences in subgroups Cleason (P < 0.001), clinical grade (P < 0.001), TNM stage (P < 0.01), suggesting the miR186 expression level is significantly associated with prostate cancer progression (Figure 7C). Moreover, Kaplan–Meier survival analysis revealed that patients with the high level of miR186 had a higher survival rate than those with the low level of miR186 (P=0.028) (Figure 7D). Thus, above data suggested that miR186 downregulation is a risk factor of prostate cancer, and decreased miR186 expression likely contributes to prostate cancer progression and might represent a prognostic biomarker for prostate cancer. On the contrary, the expression level of Twist1 was markedly increased in prostate tumor specimens (Figure 7E) and positively correlated with clinical grade (Supplementary Figure S7). The correlation analysis revealed the Twist1 levels (detected by IHC) were significantly negatively correlated with the expression levels of miR186 (measured by the real-time PCR) in clinical PCa specimens (Figure 7F). These results demonstrated that miR186 directly regulates the Twist1 level in clinical PCa specimens, indicating the potential application value of miR186 and Twist1 in the early diagnosis and treatment of PCa.

## DISCUSSION

PCa is the most common malignant tumor in men and the second highest cause of cancer mortality in developed western countries [3]. Clinically, most of advanced PCa with metastases to bone, lung or other parts of the body are correlated with poor prognosis and shorter patient survival, and metastasis is the main cause of PCa-related death in men. However, the regulatory mechanisms for metastases of PCa remain largely unknown.

Global dysregulation of miRNAs has been observed in PCa and is linked to PCa progression [23]. miR186 has dual roles either as an oncogene or a tumor suppressor gene in a certain type of cancer. For examples, miR186 inhibits proliferation by targeting cyclin D1, CDK2, and CDK6 in NSCLC cells, and miR186 downregulation is correlated with poor prognosis of NSCLC patients [24]. miR186 also inhibits the invasive activity of NSCLC cells by modulating PTTG1 expression [25]. On the other hand, miR186 as a oncogene promotes endometrial tumorigenesis by repressing the expression of tumor suppressor gene *FOXO1* [26]. In this study we found that miR186 is remarkably downregulated in malignant M12 cells and PCa specimens (especially in bone metastatic adenocarcinoma), and is negative correlated to histological grade. Thus, miR186 downregulation likely contributed to PCa pathogenesis and might represent a prognostic biomarker for PCa. In functional studies, ectopically expressed miR186 in M12 cells or knockdown of miR186 in P69 dramatically repressed or enhanced EMT, anchorage-independent growth, cell migration cell invasion, 3D culture growth and VM formation, respectively. Moreover, miR186 overexpression or knockdown in a AR-negative metastatic prostate cancer cell line PC3 or an AR-positive cell line LNCap also showed the same phenotypes as in M12/P69. Thus, these results demonstrate that miR186 plays a tumor suppressive role in PCa.

Twist1, a highly conserved basic helix–loop–helix transcription factor, is associated with different cellular processes as proliferation, EMT, cell migration/invasion, metastasis, angiogenesis, stem-cell formation, multidrug resistance and apoptosis inhibition [9, 27–29]. The high expression levels of Twist are found in many various cancer such as rhabdomyosarcoma [27], melanoma [30], pediatric osteosarcoma [31], gastric [32], breast carcinoma [28, 33]. Here we identified Twist1 as a direct target of miR186 is highly expressed in human PCa specimens and cells. Although about miR186 regulation of Twist1 expression implicating in cisplatin resistance in ovarian cancer has been recently reported [34], our data demonstrate that miR186 plays a tumor suppressive role in PCa by inhibiting tumorigenesis and metastasis, and its expression level is reversely correlated to the clinical grade and pathological grading, and the low miR186 expression is correlated with poor patient survival. Thus, our findings warrant further investigation on the potential development of miR186-based prognostic and therapeutic approaches.

## MATERIALS AND METHODS

### Cell lines and culture

P69 is a SV40-T antigen-immortalized human prostate epithelial cell line which is low-tumorigenic and non-metastatic [18], while M12 is a highly tumorigenic and metastatic subline of P69 cells generated by selection in nude mice [19, 20]. Both cell lines were cultured in RPMI 1640 supplemented with 5% fetal bovine serum (FBS), 10 ng/ml epidermal growth factor (EGF), 0.1 μM dexamethasone, 5 μg/ml insulin, 5 μg/ml transferrin, 5 ng/ml selenium and 0.05 mg/ml gentamicin. Cultures were maintained at 37°C in a 5% CO_2_ cell incubator.

### RTCA migration and RTCA invasion

Cell migration and invasion experiments were performed using the xCELLigence RTCA-DP system (Roche, Mannheim, Germany) as described previously [20, 35]. The xCELLigence System allows real time cell analysis (RTCA) by using the RTCA DP Instrument equipped with a CIM-Plate 16. The CIM-plate 16 is a 16-well system in which each well is composed of upper and lower chambers separated by an 8-μm microporous membrane. As cells migrate from the upper chamber through the membrane into the bottom chamber in response to a chemoattractant, they contact and adhere to the electronic sensors on the underside of the membrane, resulting in an increase in the electrical impedance. The increase in the impedance correlates with increasing numbers of migrated cells on the underside of the membrane. The kinetic information about the migration of cells was recorded using Real-time cell analysis and the cell index Slope (1/hr) for a time period/range was calculated according to RTCA Software Manual (Version 1.2).

Briefly, cells were serum-starved for 6 h prior to detachment for migration or invasion. Firstly, the low chamber of the CIM-Plate 16 was added 160μl of RPMI 1640 with 5% FBS, and the up chamber was added 30 μl of serum-free medium (SFM). Secondly, 4 × 10^4^ of cells (per well) were resuspended in 100 μl of SFM and loaded into the up chamber. Third, the CIM-Plate 16 containing the cells was placed onto the RTCA DP Analyzer inside the incubator at 37°C for measure at intervals of 15 minutes and the cells migrated to the low chamber were monitored. For invasion assays, the CIM-Plate 16 need to be precoated with 20 μl Matrigel (1:40 dilution, BD biosciences, Bedford, MA, USA), and after solidification 37K↜C for 4 h, cells can be seeded into the up chamber of CIM-Plate 16.

### Vasculogenic mimicry (VM) formation and 3D culture growth assays

Cells were allowed to grow to 80% confluence of monolayer. For vasculogenic mimicry assay [36], matrigel matrix^TM^ (#3445-005-01, Trevigen, Gaithersburg, MD, USA) pre-thawed at 4°C were added into the inner well of μ-slides (ibidi Gmbh, Martinsried, Germany) and incubated for at least 30 minutes at 37°C until polymerization. 50 μl of cells (1×10^5^ cells/ml) were added onto the polymerizd matrix. For 3D culture assays for invasion, 5 μl of cells (1×10^5^ cells/ml) mixed with 5 μl 3D culture matrix^TM^ were added into the inner well of μ-slides and incubated for at least 60 minutes at 37°C. After polymerization, cell-free medium were added to fill the upper well. Microscopy images were taken after three days or longer time.

### Plasmid constructions

To construct pMIR-report-Twist1-3′UTR (3′-untranslated region), the full-length Twist1-3′ UTR was amplified from human genomic DNA and cloned into the pMIR-REPORT^TM^ vector (AmBion) with SacI and HindIII restriction enzymes. The primer sequences were: Twist1-3′UTR forward atacgagctcGGAGACCTAGATGTC; Twist1-3′UTR reverse

gcgcaagcttATGCATTTAGACACC. Except the wild-type construct, the mutant one containing one putative miR186 binding site was mutated using KOD-Plus-Mutagenesis Kit (TOYOBO, Shanghai, China) with a pair of primers:

Twist1-3′UTR-MUT forward: GGCCACCCTTTT CATCCTTCCTCTGA;

Twist1-3′UTR-MUT reverse: AACTGACTATGG TTTTGCAGGCCAG.

For microRNA overexpression or knockdown, the method was described as before [37]. Briefly, the lentiviral expression vector pGreenPuro^TM^ shRNA (System Biosciences) was used to generate artificial mature miR186 for overexpression and miRZip shRNA anti-miR186 for silencing the expression of miR186. The synthetic double-stranded miR186 oligonucleotide sequences were:

miR186 forward, GATCCGCAAAGAATTCTCCT

TTTGGGCTCTTCCTGTCAGAAGCCCAAAAGCTCAATTCTTTGCTTTTTG; miR186 reverse, AATT CAAAAAGCAAAGAATTGAGCTTTTGGGCT

TCTGACAGGAAGAGCCCAAAAGGAGAATTCTTTGCG.

anti-miR186 forward,

GATCCGCAAAGAATTCAGGTTTTGGGCTCTTCCTGTCAGAAGCCCAAA

AGGAGAATTCTTTGCTTTTTG;

anti-miR-186 reverse,

AATTCAAAAAGCAAAGAATTCTCCTTTTGGGCTTCTGACAGGAAGAGCCCAAAACCTGAATTCTTTGCG.

To construct the Twist1 expression vector, the 609-bp coding sequences of Twist1 was amplified from the cDNA template of M12 cells and cloned into the lentiviral expression vector pLV-CMVenh-EGFP-CD513B (System Biosciences) with EcoRI and BamHI restriction enzymes. The primer sequences were: CD513B-Flag-Twist1 forward ggccGAATTCGCCACCATGGATTACAAGGATGACGACGATAAGATGATGCAGGACGTGTCCAG; CD513B-Flag-Twist1 reverse ATATGGATCCCTAGTGGGACGCGGA.

To construct the vector of shRNA for Twist1, the synthetic double-stranded oligonucleotides were cloned into the pLKO-puro vector (Sigma-Aldrich) using AgeI and EcoRI. The oligonucleotide sequences were:

Twist1-shRNA1-F, ccggtAGGGCAAGCGCGGCAAGAActcgagTTCTTGCCGCGCTTGCCCTtttttg

Twist1-shRNA1-R,

aattcaaaaaAGGGCAAGCGCGGCAAGAActcgagTTCTTGCCGCGCTTGCCCTa

Twist1-shRNA2-F,

ccggtGCTGGACTCCAAGATGGCAAGctcgagCTTGCCATCTTGGAGTCCAGCtttttg

Twist1-shRNA2-R,

aattcaaaaaGCTGGACTCCAAGATGGCAAGctcgagCTTGCCATCTTGGAGTCCAGCa

Twist1-shRNA3-F,

ccggtCCTGAGCAACAGCGAGGAAGActcgagTCTTCCTCGCTGTTGCTCAGGtttttg

Twist1-shRNA3-R,

aattcaaaaaCCTGAGCAACAGCGAGGAAGActcgagTCTTCCTCGCTGTTGCTCAGGa

### microRNA and mRNA quantification by real-time PCR

This method was described as before [37]. Briefly, total RNA was extracted using Trizol reagent (Invitrogen, CA, USA). 1 μg of each sample was used for reverse transcription by AMV Reverse Transcriptase (Fermentas, CA, USA), according to the manufacturer's instructions. Specific stem-loop reverse transcription primers for miRNAs were designed as described [38]. The miRNA or mRNAs levels were analyzed using the SYBR-Green Master PCR Mix (Applied Biosystems) with an ABI Stepone system (Applied Biosystems, Foster City, CA). The amplification parameters were 95°C for 10 min followed by 40 cycles of 95°C for 15 s, 55°C for 30s and 72°C for 30s. The expression of miRNA or mRNA were normalized and expressed as a percentage relative to U6 or GAPDH respectively with the following formula: fold induction = 2^[−ΔCt]^, where ΔCt = Ct_(target)_ − Ct_(U6 or GAPDH)_. Each sample was analyzed in at least triplicate. The following primer sequences were used:

Twist1 forward: GGAGTCCGCAGTCTTAC GAG,

Twist1 reverse: TCTGGAGGACCTGGTAGAGG;

GAPDH forward: AAGGTCGGAGTCAACGGA TTTG,

GAPDH reverse: CCATGGGTGGAATCATATTGG AA;

miR186 reverse transcription primer: GTCGTATCCAGTGCAGGGTCCGAGGTATTCGCACTGGATACGACAGCCCA;

miR186 forward: GCCGGCAAAGAATTCTCC TTT,

common miRNA reverse: GTGCAGGGTCCGA GGT;

U6 forward: CTCGCTTCGGCAGCACA;

U6 reverse primer: AACGCTTCACGAATTTGCGT.

### Dual luciferase reporter assay

For determination of miR186 targeting Twist1 3′-UTR, 293T cells (5×10^4^ cells per well) were plated in a 24-well plate and then co-transfected with 200 ng of either pGreenpuro-miR186 or a pGreenpuro control, 100 ng of either pMIR-REPORT^TM^ (AmBion) Firefly luciferase constructs containing the Twist1 3′-UTR WT or MUT, and 20 ng of pRL-SV40 Renilla Luciferase vector as a normalization control using Lipofectamine^TM^ 2000. 293T cells were collected 48 h after transfection and analyzed using the dual-luciferase reporter assay system (Promega, Madison, WI, USA). The pRL-SV40 vector that shows constitutive expression of Renilla luciferase was co-transfected as an internal control to correct for differences in transfection. Transfections were performed in triplicate and repeated at least three independent times.

### Soft-agar colony formation assay

The ability of anchorage-independent growth was evaluated by a soft agar assay as described [39]. Briefly, 3×10^3^ cells of each clone were suspended in culture medium containing 5% with 0.35% Bacto agar (Amresco, OH, USA). The agar cell mixture was plated on top of a bottom layer with 0.6% agar-medium mixture in six-well plates. After 14 ~18 days, cell colonies were fixed and stained with 1 ml of 0.005% Crystal Violet for 1 hour. The photographs of the cells growing in the plate and the colonies developed in soft agar were taken, the number of colonies larger than 0.5 mm was scored by ImageJ V1.45 (NIH, USA).

### Wound healing assays for migration

This method for analysis of migration was conducted as described previously [36]. Briefly, 1~2 ×10^4^ of cells were plated into the μ-Dish (35mm high, ibidi Gmbh, Martinsried, Germany) and cultured overnight to ensure adhered. A clear area was created by removing the Culture-Insert from the μ-Dish, and photos were taken as indicated time until the wound was healed.

### microRNA *in situ* hybridization (ISH)

Prostate cancer tissue arrays purchased from ALenabio (Cat#PR956, Xi'an, China) were deparaffinized with xylene (5 minutes, repeated 3 times), washed with 70%, 95% and 100% of ethanol (each for 3 times) and by two changes of DEPC-PBS buffer. Tissues were then digested with proteinase K (15 μg/ml) for 20 minutes at 37°C, rinsed 3 times with DEPC-PBS, and then air-dried.

The above pretreated tissue array slides were hybridized with Digoxigenin-conjugated oligonucleotide probes for human hsa-miR186 (40 μM) for 1 hour at 37°C or at 4°C overnight, and incubated with DNase for 30 minutes at 37°C, rinsed 3 times with DEPC-PBS buffer. Following hybridization, the array slides were rinsed twice with 5×SSC, 2×SSC and 1×SSC for 5 minutes. The slides were incubated with blocking solution for 15 minutes and then with anti-DIG antibody (1:800) in 2% sheep serum blocking solution for 1 hour at 25°C. Following washes 3 times with PBS-T (PBS, 0.1%Tween-20), slides were incubated with the alkaline phosphatase (AP) substrate (NBT-BCIP) in 10 ml of 0.2 mM Levamisole for 2 hour at 30°C in the dark. The reaction was stopped with 2 washes of AP stop solution (50 mM Tris-HCl, 150 mM NaCl, 10 mM KCl) and 2 washes with double-distilled water. Tissue slides were counter stained with Nuclear Fast Red for 1 minute and rinsed with double-distilled water. Slides were dehydrated twice with 70%, 95% and 100% of ethanol and mounted with coverslips in Eukitt mounting medium. All images were captured and processed using identical settings.

### Immunohistochemical staining (IHC)

This method for analysis of migration was conducted as described previously [20]. Briefly, human prostate tissue array slides were stained with antibody against Twist1 mAb at 4°C overnight and then with secondary antibody conjugated with HRP at 37°C for 30 min. The peroxidasecatalyzed product was visualized with the DAB Chromogen Kit (brown) followed by hematoxylin counterstain (blue). The threshold of Twsit1-positive hue was determined by several control fields which were optimal separation between brown and blue stained areas and data were presented by the ratio of Twist1 stained positive area/total area.

### Hematoxylin and eosin staining (H&E)

Paraffin-embedded sample preparation, hematoxylin and eosin staining (H&E) were performed as previously described [39].

### *In vivo* metastasis experiments

PC3^Luc^ cells [39] stably expressing the empty vector, miR186, miR186-CD513B (vector), or miR186+Twist1 were used in *in vivo* metastasis assays. 1.0×10^6^ of each cell lines in 100 μl phosphate-buffered saline were respectively injected into the left cardiac ventricle of BALB/c nude male mice at 7 weeks old by intracardiac injection. Two weeks after the injection, the metastasis nodules was monitored every two weekly by bioluminescence imaging. Mice were anesthetized and given intraperitoneal injection of D-luciferin and 10 minutes after the injection, the bioluminescence images were taken with a charge-coupled device camera (IVIS, Xenogen). Bioluminescence of the tissue metastases were quantified using the Living Image software. Six weeks later, the mice were killed and the metastatic nodules were dissected according to the images. Paraffin-embedded sections were then prepared and subjected to Hematoxylin and eosin staining (H&E staining). All animal studies were conducted with the approval and guidance of Shanghai Jiao-Tong University Medical Animal Ethics Committees.

### Statistical analysis

All statistical analyses were carried out using the SPSS 18.0 statistical software package. Comparisons of the miR186 levels in subgroups (clinical grade, Cleason, TNM stage) for significance were analyzed by one-way ANOVA. 38 of PCa patients (missing 2 patient's data) were divided into 2 groups based on the miR186 expression level, the miR186-low group (less than the median value) and the miR186-high group (more than the median value) for clinical survival analysis. Survival curve was plotted using the Kaplan–Meier survival curve and analyzed by the log-rank test. The correlation between the miR186 and Twist1 levels in human clinical PCa specimens was analyzed by Pearson Correlation test and linear correlation and regression. Comparisons between groups for statistical significance were conducted with a 2-tailed Student's t-test. P<0.05(*), P<0.01(**) and P<0.001(***) were considered statistically significant in all cases.

## SUPPLEMENTARY FIGURES AND TABLE


