# Supplementary material for: miR186 suppresses prostate cancer progression by targeting Twist1
Source: Oncotarget. 2016 Apr 21;7(22):33136–51. doi: 10.18632/oncotarget.8887 (PMC5078081; doi:10.18632/oncotarget.8887)
Supplement: Supplementary file 1 [file oncotarget-07-33136-s001.pdf]

SUPPLEMENTARY FIGURES AND TABLE

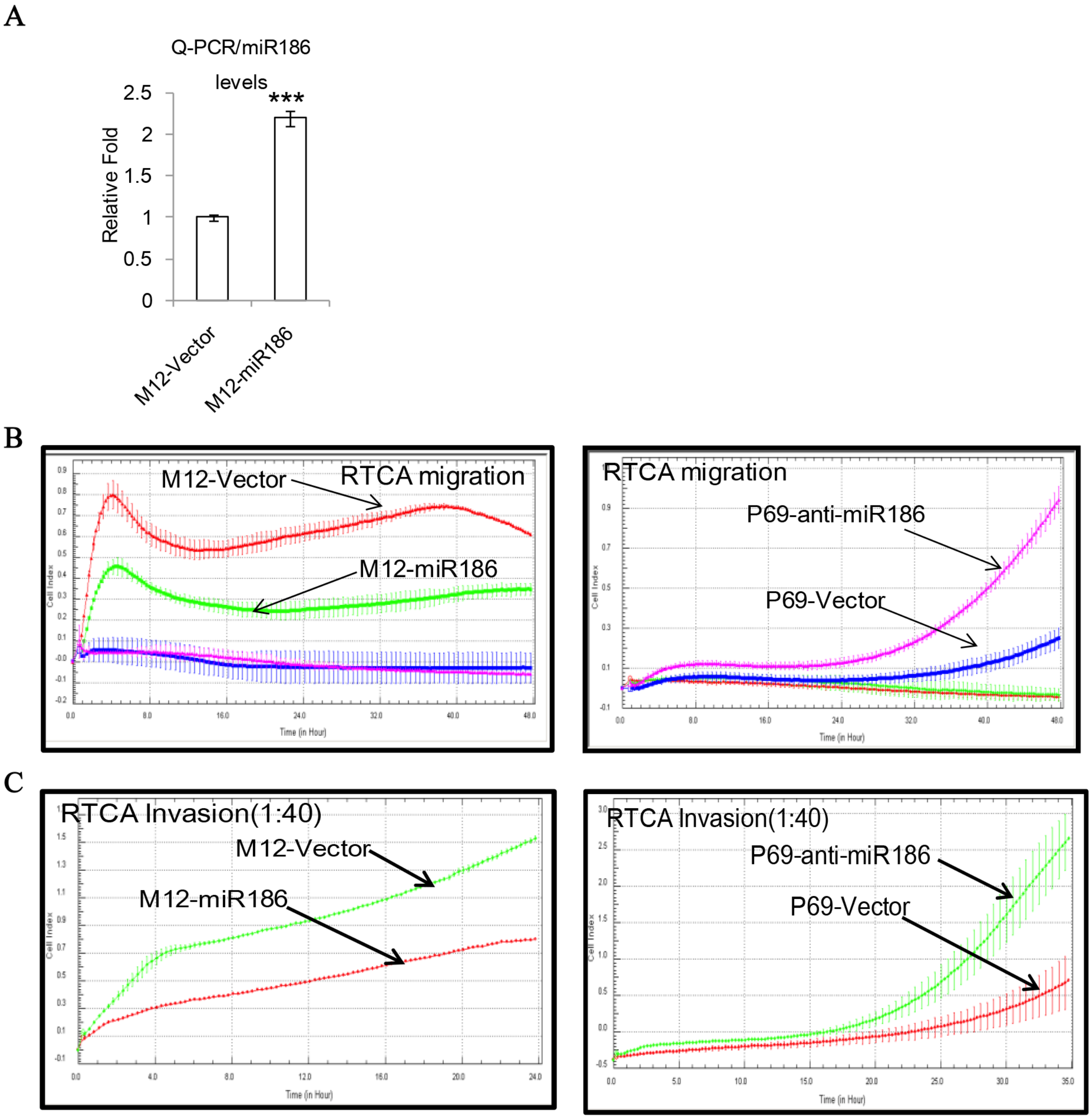

Supplementary Figure S1: miR186 suppresses PCa cell migration and invasion. A. The expression levels of miR186 in M12 cells stably transfected with the control vector or miR186 were measured by stem-loop real-time PCR. P-values of < 0.001 (***). **B–C.** RTCA monitoring of cell migration (B) or invasion (C). M12-vector and M12-miR186 or P69-vector and P69-anti-miR186 cells were seeded into a CIM-Plate without or with pre-coated matrigel (1:40) and subjected to a dynamic analysis lasting for 48 h, respectively. The migration or invasion curve was shown as histogram. Error bars indicate ±SD, *P*-values of < 0.001 (***) (These are related to Figure 2C–2D).

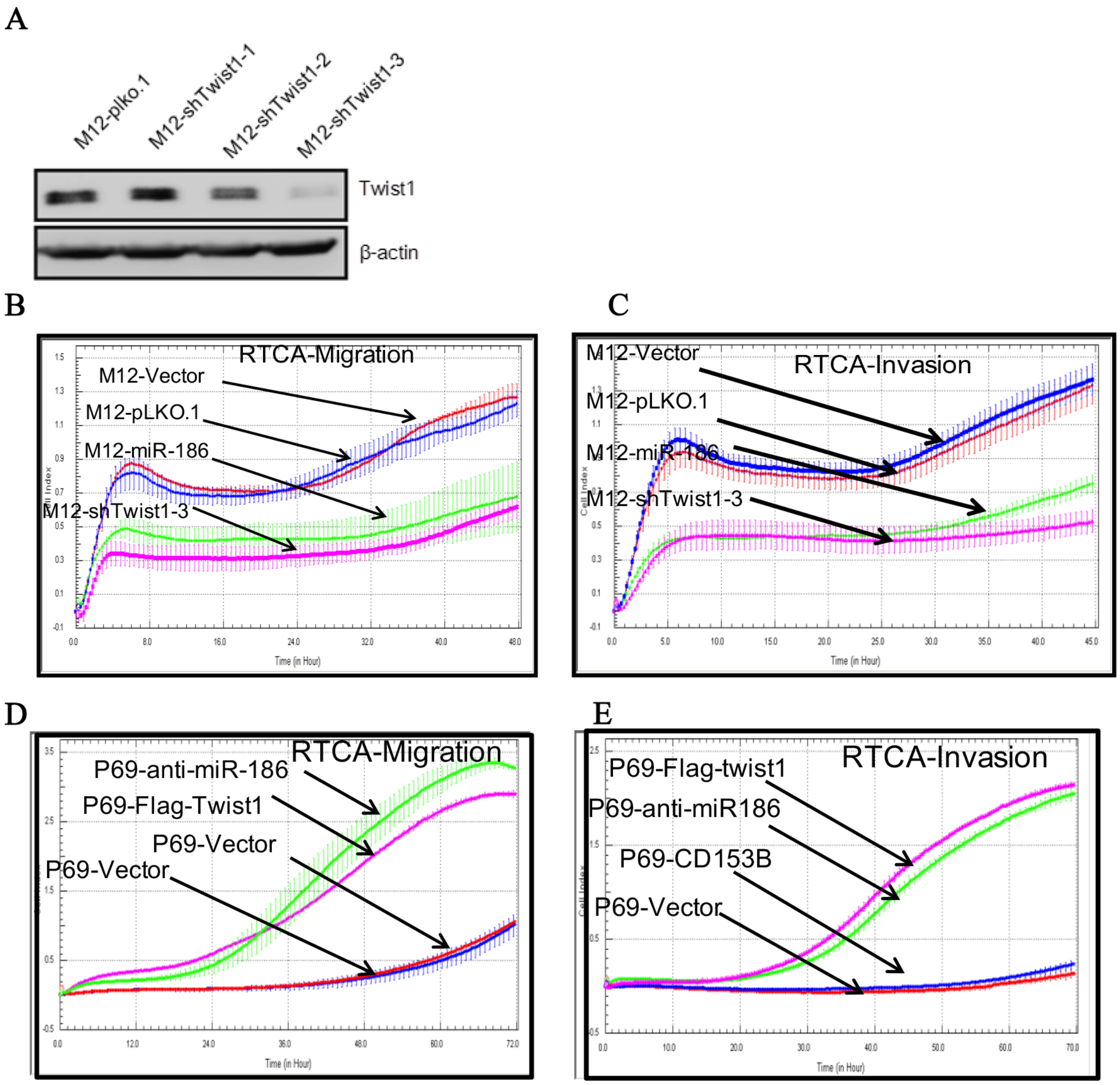

Supplementary Figure S2: Twist1 mimics miR186-mediated phenotypes in PCa cells. A. The levels of Twist1 in M12 cells stably transfected with the lenti-vector pLKO.1 or shTwist1(1/2/3) were analyzed by Western blotting. **B–E.** RTCA monitoring of cell migration (B, D) or invasion (C, E) using the xCELLigence system. Stable M12 group (M12-vector, M12-miR186, M12-pLKO.1 and M12-shTwist1-3 in B–C) or P69 group (P69-vector, P69-anti-miR186, P69-CD513B, and P69-Flag-Twist1 in C–E) cells were seeded to a CIM-Plate without or with pre-coated matrigel (1:40) and subjected to a dynamic analysis lasting for 48 or 72 h, respectively. The migration or invasion curve was shown as histogram. Error bars indicate ±SD, P-values of < 0.001 (***). (These are related to Figure 4B, 4C, 4F, 4G).

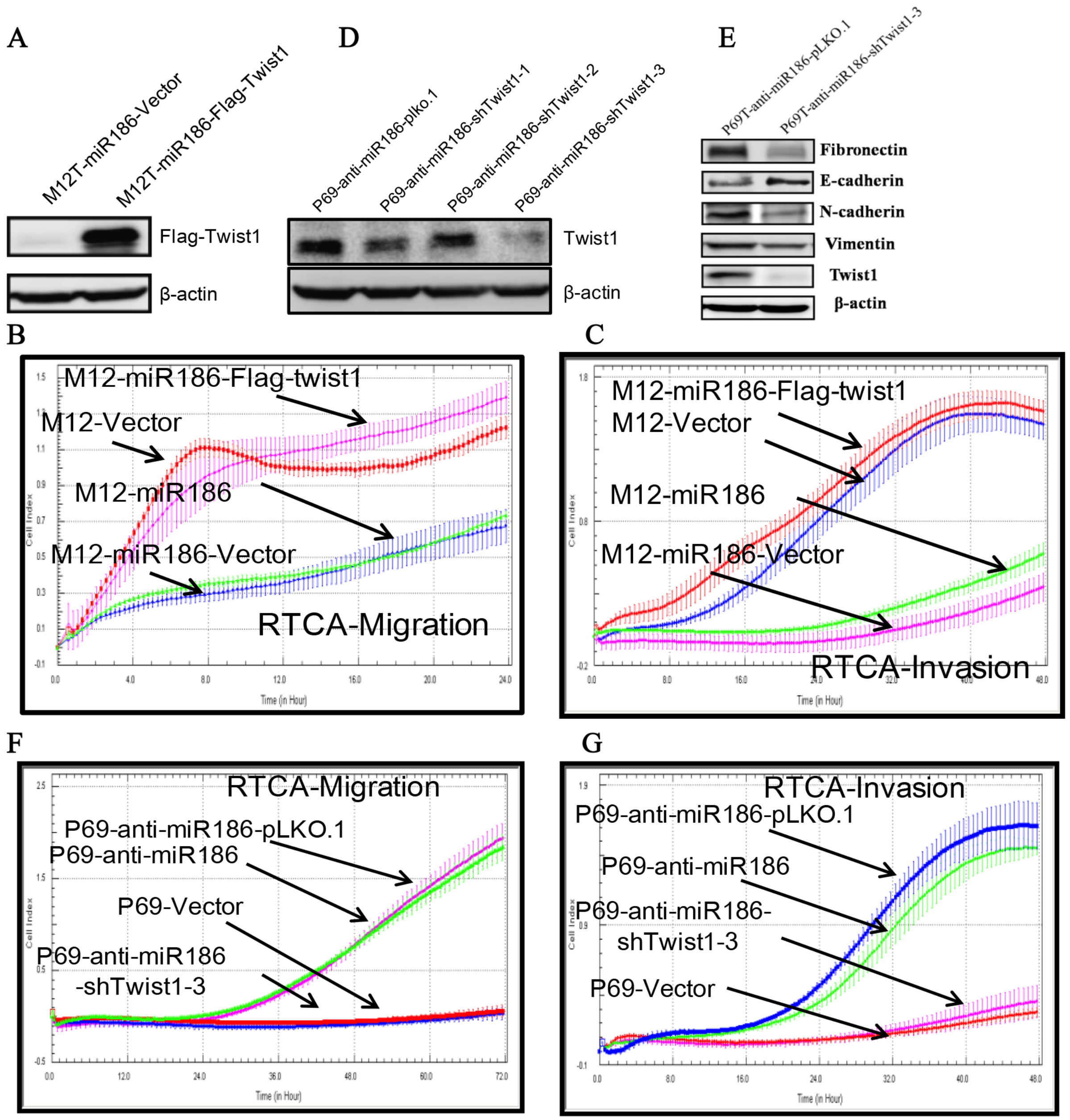

Supplementary Figure S3: miR186 functions by downregulation of Twist1 in PCa cells. A, D, E. Western blotting analysis for the levels of Twist1 in M12-miR186 cells stably transfected with vector or Flag-twist1 (A) or in P69-anti-miR186 cells stably transfected with pLKO.1 vector or shTwist1-1/2/3 (D) and for epithelial and mesenchymal markers in P69-anti-miR186 cells stably transfected with pLKO.1 vector or shTwist1-3 (E). **B–C, F–G.** RTCA monitoring of cell migration (B, F) or invasion (C, G). M12 group (M12-Vector, M12-miR186, M12-miR186-CD513B and M12-miR186-Twist1 in B–C) or P69 group (P69-Vector, P69-anti-miR186, P69-miR186-pLKO.1 and P69-anti-miR186-shTwist1-3 in F–G) cells were seeded into a CIM-Plate without or with pre-coated matrigel (1:40) and subjected to a dynamic analysis lasting for 48 or 72 h, respectively. The migration or invasion slope was shown as histogram. Error bars indicate ±SD, *P*-values of < 0.001 (***). (These are related to Figure 5B, 5C, 5G, 5H).

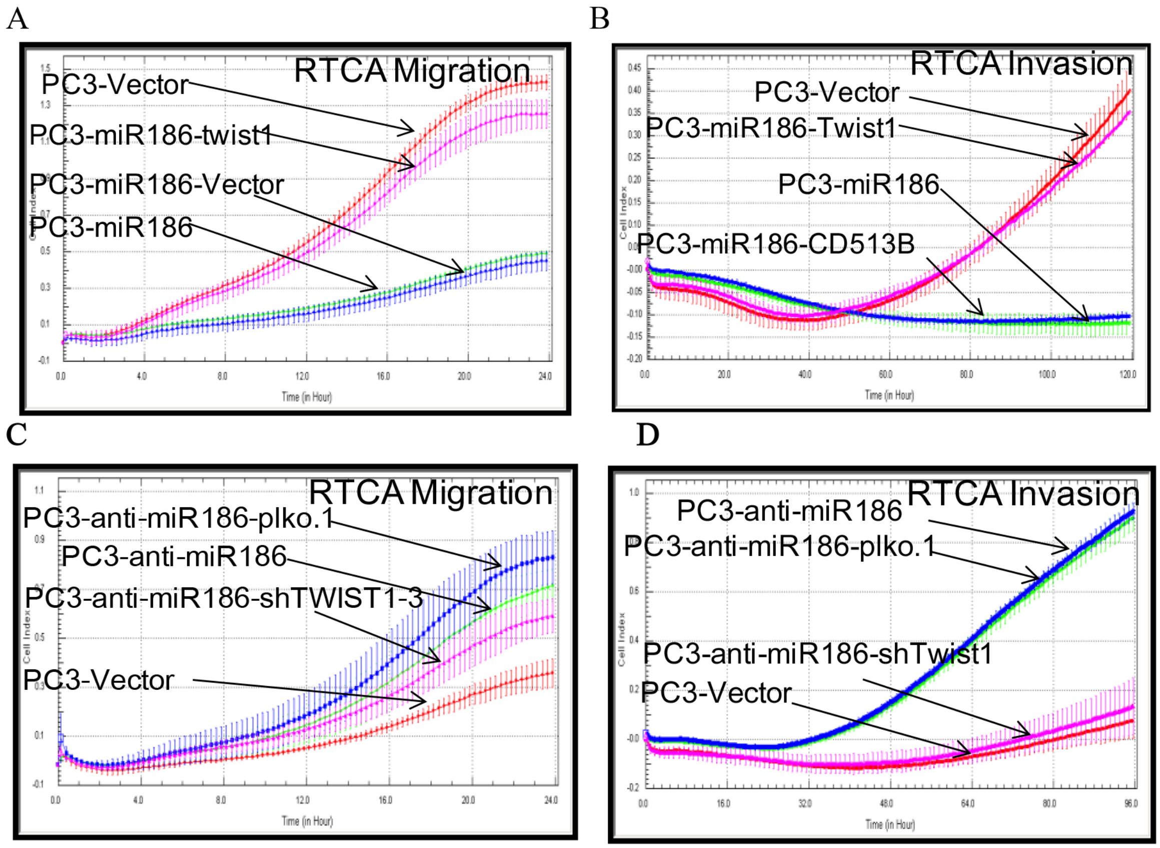

Supplementary Figure S4: The miR186-Twist1 axis suppresses PCa cell migration and invasion. RTCA monitoring of cell migration **A, C.** or invasion **B, D.** One group (PC3-Vector, PC3-miR186, PC3-miR186-CD513B and PC3-miR186-Twist1) and another group (PC3-Vector, PC3-anti-miR186, PC3-anti-miR186-pLko.1 and PC3-anti-miR186-shTwist1) cells were seeded into a CIM-Plate without or with pre-coated matrigel (1:40) and subjected to a dynamic analysis lasting for 48 or 96 h, respectively. The migration or invasion slope was shown as histogram. Error bars indicate ±SD, *P*-values of < 0.001 (***). (These are related to Figure 5B, 5C, 5E, 5F).

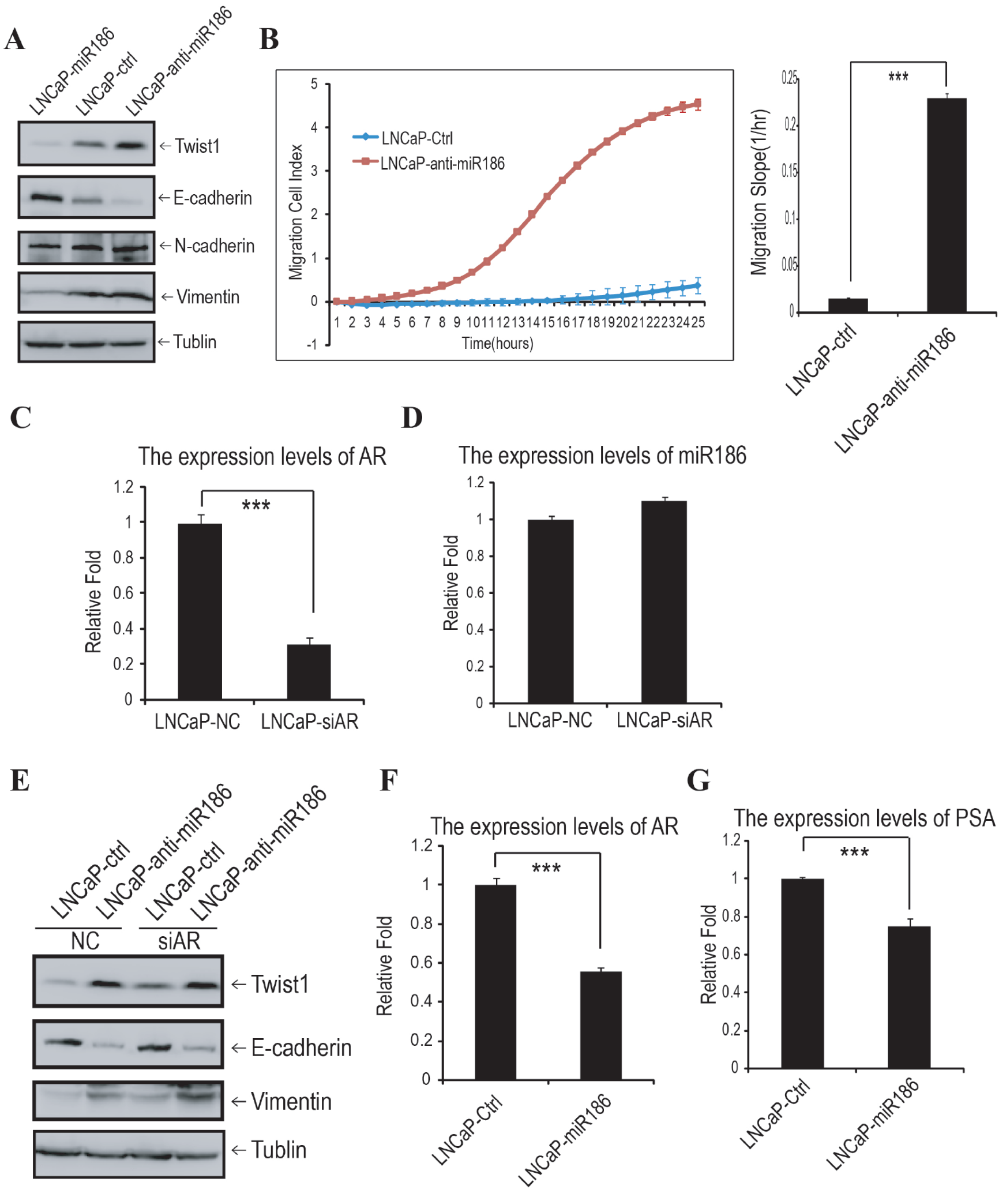

Supplementary Figure S5: The miR186-Twist1 axis in the AR-positive cell line LNCaP. A, E. Immunoblotting of epithelial/ mesenchymal markers and Twist1 in LNCaP-Ctrl (Empty vector), LNCaP-miR186 and LNCaP-anti-miR186 cells (A) or in LNCaP-Ctrl and LNCaP-anti-miR186 cells with or without siRNA-AR (siAR) transfection (E). **B.** RTCA monitoring of cells migration. LNCaP-Ctrl and LNCaP-anti-miR186 cells were seeded to a CIM-Plate and subjected to a dynamic migration assay lasting for 25 h. The cell migration curve (left panel) and slope (right panel) were shown. Error bars indicate ±SD., *P*-values of < 0.001 (***). **C–D, F–G.** The levels of AR mRNA (C) and miRNA186 (D) in LNCaP cells transfected with NC (control siRNA) or siAR were measured by qRT-PCR. The mRNA levels of *AR* (F) and *PSA* (G) in LNCaP-Ctrl and LNCaP-miR186 cells were measured by qRT-PCR. Error bars indicate represent the mean ± SEM. of three independent experiments with triplicates each, *P*-values of < 0.001 (***).

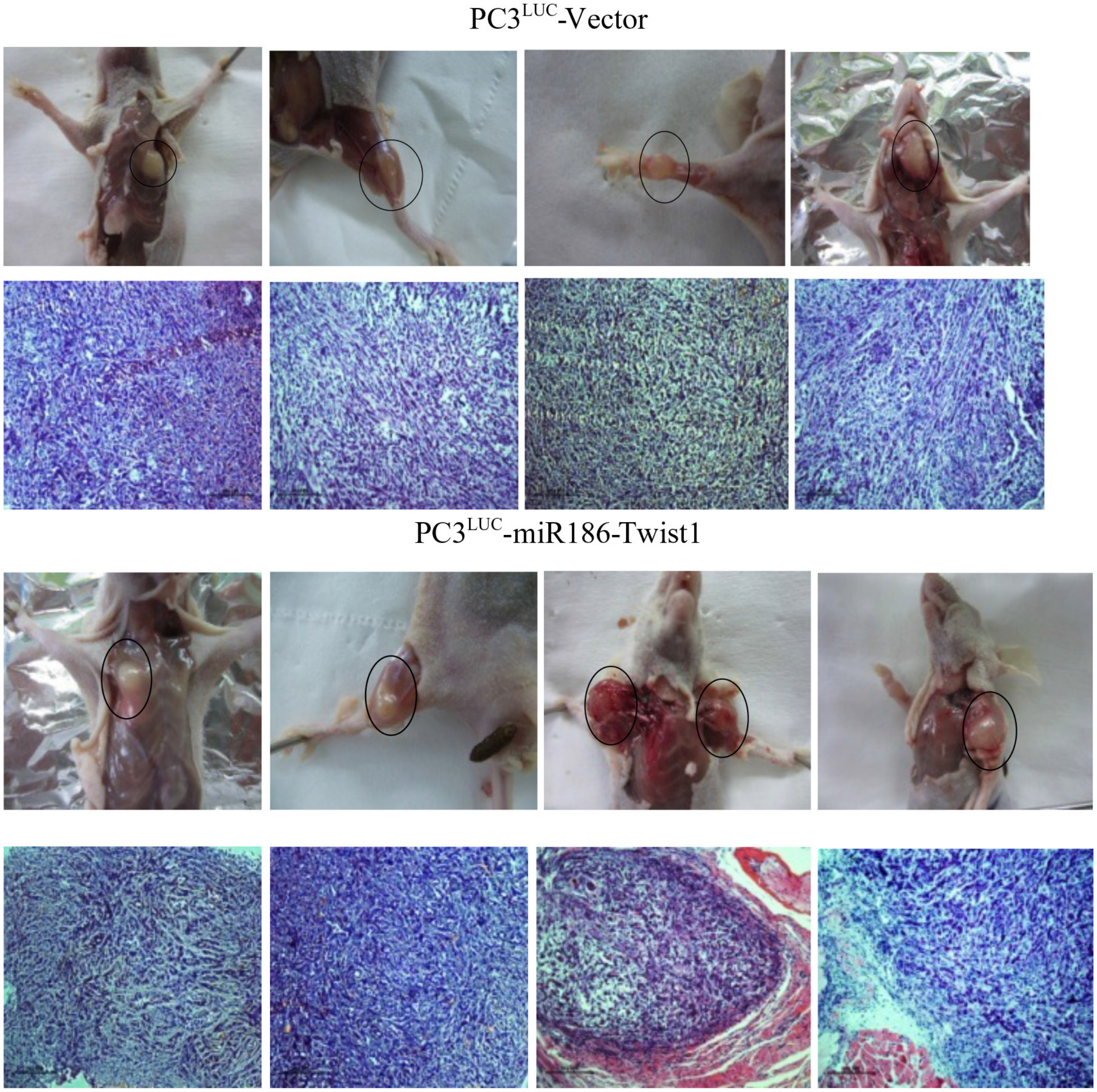

Supplementary Figure S6: The miR186-Twist1 axis suppresses prostate cancer metastasis *in vivo*. (These are related to Figure 6H). 1.0×10^6 of stable cell lines $PC3^{Luc}$-Vector, $PC3^{Luc}$-miR186, $PC3^{Luc}$-miR186-Vector and $PC3^{Luc}$-miR186-Twist1 were respectively injected into the left cardiac ventricle of BALB/c nude mice at 7 weeks old by intracardiac injection. Mice were killed 6 weeks later, and tumors were dissected and stained with H&E staining.

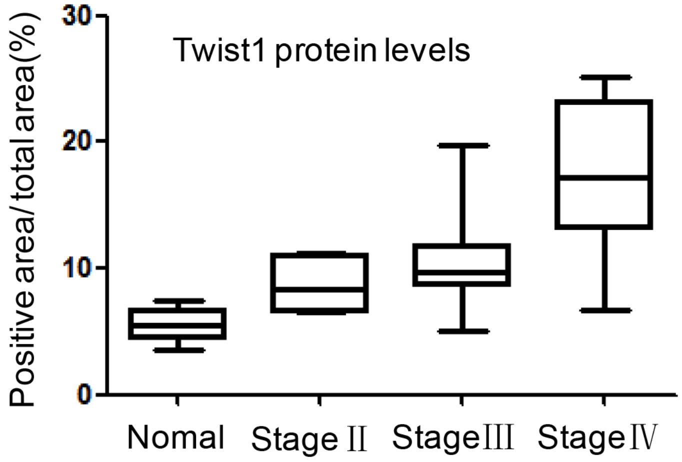

Supplementary Figure S7: Negative correlation between miR186 and Twist1 in PCa clinical specimens. (These are related to Figure 7E). Assessment of Twist1 levels by immunohistochemistry in normal prostate tissues and prostate carcinomas according to the tumor stages.

Supplementary Table S1: Characteristics of clinical patients with prostate cancer

No.	Age	Organ	Pathological diagnosis	Grade	Gleason	TNM	PSA (ng/ml)	
1	68	Prostate	Adenocarcinoma	III	9	T3bN0M0	-	Malignant
2	64	Prostate	Adenocarcinoma	II	7	T2cN0M0	30	Malignant
3	71	Prostate	Adenocarcinoma	III	9	T3bN0M0	60.4	Malignant
4	64	Prostate	Adenocarcinoma	III	10	T3aN0M0	7.4	Malignant
5	59	Prostate	Adenocarcinoma	III	9	T3bN0M0	9.8	Malignant
6	65	Prostate	Adenocarcinoma	IV	8	T4N0M0	34.9	Malignant
7	73	Prostate	Adenocarcinoma	II	7	T2cN0M0	48.1	Malignant
8	69	Prostate	Adenocarcinoma	II	7	T2cN0M1	10.6	Malignant
9	62	Prostate	Adenocarcinoma	II	7	T2cN0M1	37.3	Malignant
10	66	Prostate	Adenocarcinoma	III	9	T3bN0M0	1.2	Malignant
11	60	Prostate	Adenocarcinoma	III	9	T3bN0M0	40	Malignant
12	70	Prostate	Adenocarcinoma	IV	7	T4N0M0	7	Malignant
13	65	Prostate	Adenocarcinoma	III	9	T3bN0M1	17.5	Malignant
14	67	Prostate	Adenocarcinoma	IV	9	T3bN1M0	13.1	Malignant
15	69	Prostate	Adenocarcinoma	III	7	T3bN0M0	1.1	Malignant
16	69	Prostate	Adenocarcinoma	III	7	T3aN0M1	17.6	Malignant
17	70	Prostate	Adenocarcinoma	III	7	T3aN0M1	9	Malignant
18	58	Prostate	Adenocarcinoma	III	9	T3bN0M0	5.8	Malignant
19	71	Prostate	Adenocarcinoma	II	7	T2cN0M0	31.4	Malignant
20	70	Prostate	Adenocarcinoma	III	7	T3bN0M0	14.4	Malignant
21	59	Prostate	Adenocarcinoma	II	6	T2bN0M0	18.3	Malignant
22	63	Prostate	Adenocarcinoma	III	9	T3bN0M0	16.6	Malignant
23	72	Prostate	Adenocarcinoma	III	9	T3bN0M0	-	Malignant
24	66	Prostate	Adenocarcinoma	III	8	T3bN0M0	10.8	Malignant
25	70	Prostate	Adenocarcinoma	II	7	T2cN0M1	-	Malignant
26	68	Prostate	Adenocarcinoma	III	8	T3bN0M0	26.9	Malignant
27	63	Prostate	Adenocarcinoma	III	10	T3bN0M1	-	Malignant
28	57	Prostate	Adenocarcinoma	III	7	T3bN0M0	25	Malignant
29	72	Prostate	Adenocarcinoma	III	8	T2cN0M0	16.8	Malignant
30	70	Prostate	Adenocarcinoma	III	8	T3bN0M0	0.5	Malignant
31	75	Prostate	Adenocarcinoma	III	9	T3bN0M0	98	Malignant
32	62	Prostate	Adenocarcinoma	III	9	T3bN0M0	-	Malignant
33	63	Prostate	Adenocarcinoma	III	9	T3bN0M0	91	Malignant
34	53	Prostate	Adenocarcinoma	III	9	T3bN0M0	161	Malignant
35	63	Prostate	Adenocarcinoma	III	8	T3bN0M0	13	Malignant

(*Continued*)

No.	Age	Organ	Pathological diagnosis	Grade	Cleason	TNM	PSA (ng/ml)	
36	44	Prostate	Adenocarcinoma	III	7	T3bN0M1	-	Malignant
37	65	Abdominal wall	Metastatic adenocarcinoma from prostate	IV	.	M1	-	Metastasis
38	61	Bone	Metastatic adenocarcinoma from prostate	IV	.	M1	-	Metastasis
39	69	Bone	Metastatic adenocarcinoma from prostate	IV	.	M1	-	Metastasis
40	59	Bone	Metastatic adenocarcinoma from prostate	IV	.	M1	-	Metastasis
41	69	Prostate	Normal (match of #8)	.	.	.	-	NAT
42	62	Prostate	Normal (match of #9)	.	.	.	-	NAT
43	65	Prostate	Normal (match of #13)	.	.	.	-	NAT
44	69	Prostate	Normal (match of #16)	.	.	.	-	NAT
45	70	Prostate	Normal (match of #17)	.	.	.	-	NAT
46	70	Prostate	Normal (match of #25)	.	.	.	-	NAT
47	63	Prostate	Normal (match of #27)	.	.	.	-	NAT
48	44	Prostate	Normal (match of #36)	.	.	.	-	NAT

*The information of TNM and stage is according to AJCC Cancer Staging Manual (6th Edition),
**The grade 1-3 (or I-III) in Pathology Diagnosis is equivalent to well-differentiated,moderately-differentiated or poorly differentiated, respectively, under microscope.
Grade 1 or well-differentiated: Cells appear normal and are not growing rapidly.
Grade 2 or moderately-differentiated: Cells appear slightly different than normal.
Grade 3 or poorly differentiated: Cells appear abnormal and tend to grow and spread more aggressively.
Grade 4 or undifferentiated: *(for certain tumors), features are not significantly distinguishing to make it look any different from undifferentiated cancers which occur in other organs.
***TNM grading:
T - Primary tumor
Tx - Primary tumor cannot be assessed
T0 - No evidence of primary tumor
Tis - Carcinoma in situ; intraepithelial or invasion of lamina propria
T1 - Tumor invades submucosa
T2 - Tumor invades muscularis propria
T3 - Tumor invades through muscularis propria into subserosa or into non-peritonealized pericolic or perirectal tissues.
T4 - Tumor directly invades other organs or structures and/or perforate visceral peritoneum
N - Regional lymph nodes
Nx - Regional lymph nodes cannot be assessed
N0 - No regional lymph node metastasis
N1 - Metastasis in 1 to 3 regional lymph nodes
N2 - Metastasis in 4 or more regional lymph nodes
M - Distant metastasis
Mx - Distant metastasis cannot be assessed
M0 - No distant metastasis
M1 - Distant metastasis
